# Oxidative Stress in the Newborn Period: Useful Biomarkers in the Clinical Setting

**DOI:** 10.3390/antiox7120193

**Published:** 2018-12-14

**Authors:** Iván Millán, José David Piñero-Ramos, Inmaculada Lara, Anna Parra-Llorca, Isabel Torres-Cuevas, Máximo Vento

**Affiliations:** 1 Neonatal Research Group, Health Research Instituto La Fe, 46026 Valencia, Spain; imiya@alumni.uv.es (I.M.); josedavidpineiro@gmail.com (J.D.P.-R.); inmb612@gmail.com (I.L.); annaparrallorca@gmail.com (A.P.-L.); maximo.vento@uv.es (M.V.); 2 Division of Neonatology, University and Polytechnic Hospital La Fe, 46026 Valencia, Spain

**Keywords:** oxygen, oxidative stress, biomarkers, preterm, hypoxia, reoxygenation, antioxidant system, reactive oxygen species, mass spectrometry

## Abstract

Aerobic metabolism is highly efficient in providing energy for multicellular organisms. However, even under physiological conditions, an incomplete reduction of oxygen produces reactive oxygen species and, subsequently, oxidative stress. Some of these chemical species are highly reactive free radicals capable of causing functional and structural damage to cell components (protein, lipids, or nucleotides). Oxygen is the most used drug in ill-adapted patients during the newborn period. The use of oxygen may cause oxidative stress-related diseases that increase mortality and cause morbidity with adverse long-term outcomes. Conditions such as prematurity or birth asphyxia are frequently treated with oxygen supplementation. Both pathophysiological situations of hypoxia–reoxygenation in asphyxia and hyperoxia in premature infants cause a burst of reactive oxygen species and oxidative stress. Recently developed analytical assays using mass spectrometry have allowed us to determine highly specific biomarkers with minimal samples. The detection of these metabolites will help improve the diagnosis, evolution, and response to therapy in oxidative stress-related conditions during the newborn period.

## 1. Introduction

Fetal life occurs in a relatively hypoxic environment. Hypoxia is necessary for the development and growth of the fetus. Under normal circumstances, the fetal-to-neonatal transition causes physiological oxidative stress (OS), which enhances the antioxidant defense and pulmonary surfactant maturation [1]. Premature babies have both immature lungs and antioxidant defense systems and often require oxygen supplementation to overcome respiratory distress. The combination of hyperoxia, which enhances the generation of reactive oxygen species (ROS), and low antioxidants cause oxidative stress, inflammation, and even apoptosis, thus increasing mortality and morbidity [2]. Birth asphyxia is characterized by successive periods of hypoxia reoxygenation. Experimental studies in animal models and clinical studies have unraveled the pathophysiologic mechanisms triggered during hypoxia–reoxygenation. During hypoxia, there is an accumulation of purine derivatives. Of note, during reoxygenation, there is a generalized activation of oxidases, especially xanthine oxidase, which leads to the formation of a burst of ROS that cause tissue damage. Specific molecular biomarkers that reflect the tissue injury in different neonatal conditions, such as bronchopulmonary dysplasia (BPD), retinopathy of prematurity (ROP), or hypoxic-ischemic encephalopathy (EHI), have been identified. Analytical methods are based in the use of ultra-performance liquid chromatography coupled with tandem mass spectrometry (UPLC-MS/MS) or gas chromatography–mass spectrometry (GS-MS/MS). These techniques are highly sensitive and selective, need minimal samples, and can be adapted to different matrices, such as plasma, urine, or amniotic fluid. At present, they constitute the most powerful tool for the assessment of oxidative damage with a wide array of applications in neonatal medicine [3].

This review aims to inform the reader about the OS inherent to the fetal-to-neonatal transition under physiologic and pathologic conditions and describe recently developed analytical methods to accurately determine OS biomarkers that aid to better diagnose and treat perinatal conditions.

## 2. Aerobic Metabolism, Reactive Oxygen Species, and Oxidative Stress

**Figure 1:**
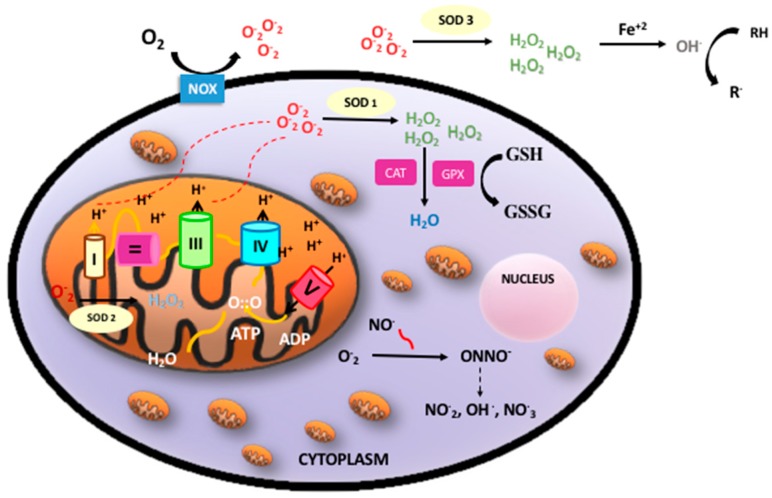
Oxidative phosphorylation, reactive oxygen species (ROS)/reactive nitrogen species (RNS), and antioxidant defense. Oxidative phosphorylation in the mitochondria builds up adenosine triphosphates (ATP) from highly energized electrons from the Krebs’ cycle. The antioxidant system: Superoxide anion (O_2_^−^) mainly produced in the mitochondria is dismutated to hydrogen peroxide (H_2_O_2_) by superoxide dismutase (SOD1 intracellular, SOD2 mitochondrial, SOD3 extracellular). H_2_O_2_ maybe detoxified by both catalase (CAT) or glutathione peroxidase (GPX) producing water (H_2_O). In addition, free radicals can be neutralized by electrons donated by reduced glutathione (GSH) when converted into oxidized glutathione (GSSG) with the concourse of a set of enzymes under the common denominator of “glutathione redox cycle enzymes”, which comprise GPX, glutathione reductase (GRD), and glutathione S-methyl transferase (GP-S-methyl).

Oxidative phosphorylation is a metabolic process by which the respiratory enzymes of the mitochondria synthesize adenosine triphosphates (ATP) during the oxidation and ulterior phosphorylation of organic molecules, such as carbohydrates, fatty acids, or amino acids. Oxidation is an essential part of both aerobic life and metabolism, because it provides energy for metabolism, growth, and the development of multicellular organisms [4]. Di-oxygen (O_2_) has paramagnetic properties and, therefore, undergoes a slow reduction process to acquire the four electrons needed to stabilize its outer orbit. However, in the presence of transition metals (e.g., iron, copper, zinc, manganese, and selenium), oxygen turns into highly reactive free radicals, which rapidly oxidize when molecules convert them into free radicals and alter their structure and function [5]. Molecular di-oxygen reduced with just one electron is converted into the superoxide anion radical (O_2_^−^); a second one-electron reduction with concomitant acceptance of two protons yields hydrogen peroxide (H_2_O_2_). H_2_O_2_ that accepts one more electron is split up into the hydroxyl radical (OH). Therefore, incompletely reduced O_2_ leads to the formation of ROS [6]. In addition, nitric oxide (NO) may combine with oxygen free radicals, especially the superoxide anion, to form peroxynitrite (ONOO^−^), a reactive nitrogen species (RNS). ROS and RNS rapidly oxidize nucleic acids, lipids, and proteins, thereby altering their structure and function and modifying the normal cellular redox status, leading to increased OS [7] (Figure 1).

ROS are generated in several intracellular compartments, such as the plasma membrane, the peroxisomes, the endoplasmic reticulum, and in the cytosol. The main endogenous source of ROS is the mitochondrial electron transport chain. In addition, free circulating transition metals (Fenton reaction) and enzymes that catalyze ROS-generating chemical reactions, such as peroxidases, NADPH oxidase, xanthine oxidase, lipoxygenases, myeloperoxidase, nitric oxide synthase, and cyclooxygenases, are also relevant sources of ROS [8]. However, there are circumstances such as infection, radiation, or inflammation in which other subcellular structures such as peroxisomes, Golgi apparatus, or endoplasmic reticulum become sources of ROS that outweigh the mitochondria (for a review see [9]).

At low concentrations, ROS and RNS play important roles as regulatory mediators in signaling processes, whereas, at moderate or high concentrations, they are harmful for living organisms, altering cellular function and the redox balance, which is indispensable for reproduction, growth, and differentiation [10]. The redox system may modify the functions of proteins through regulation of their expression, post-translational modifications, and stability. H_2_O_2_ can also act as a cell-signaling molecule that regulates the redox-mediated oxidation of cysteine residues within proteins [11]. Cysteine residues exposed on the surfaces of proteins are the dominant intracellular thiol and play an important role in intracellular antioxidant defense and the redox regulation code [12]. OS is a consequence of the interruption of these circuits by different mechanisms [13,14].

## 3. The Fetal-to-Neonatal Transition

### 3.1. Cardiocirculatory and Metabolic Changes during Birth and Postnatal Stabilization

The fetal-to-neonatal transition implies a series of complex changes that lead to the establishment of air breathing and the closure of intra- and extracardiac shunting, coinciding with the clamping of the umbilical cord [15,16]. Within a few minutes after birth, the partial pressure of oxygen (PaO_2_) increases from 3.3 kPa (25–35 mmHg) to 10.5 kPa (80–90 mmHg) [3], and the oxygen saturation (SpO_2_) plateaus between 95% and 100% at around 5 min in full-term infants and 7–8 min in those born prematurely [17]. The abrupt increase in oxygen availability to the tissue causes physiologic OS with the generation of ROS. Under normal conditions, OS contributes to the activation of enzymatic pathways that facilitate neonatal adaptation [18]. However, asphyxiated newborn infants or very premature infants (<32 weeks of gestation) need positive pressure ventilation with oxygen supplementation to be successfully stabilized. This resuscitation can be effective, although such life-saving measures will generate great amounts of free radicals [19]. It has been shown that very premature infants with gestational ages of <32 weeks of gestation have lower SOD and CAT activities [20] and lower ability to synthesize glutathione [21], and, therefore, such premature infants are ill-prepared to face OS [3].

### 3.2. Experimental Models of Oxidative Stress in the Fetal-to-Neonatal Transition

Oxidative stress is inherent to the fetal-to-neonatal transition under physiologic circumstances. The reduced glutathione to oxidized glutathione ratio (GSH/GSSG) was compared in isolated hepatocytes of fetal, offspring, and adult Whistar rats after normal pregnancies and deliveries. The GSH/GSSG ratio was significantly lower in the offspring as compared with the fetal and adult rats [22]. GSH/GSSG is the most relevant marker of global oxidative stress, particularly cytoplasmic redox status [23]. The administration of N-Acetyl-Cysteine increased GSH synthesis in pregnant Whistar rats and prevented OS associated with the fetal-to-neonatal transition in the offspring [24]. Gelfand et al. showed that the use of pure oxygen after a hypoxic episode in intubated and ventilated rat pups contributed to significantly reduce the GSH/GSSG ratio, causing intense OS in the brain and the liver [25]. In addition, in a murine model of hypoxia–reoxygenation where rat pups were recovered with oxygen concentrations of between 21% and 100%, Presti et al. demonstrated that there were no differences in the short- and long-term outcomes. Although the use of 100% oxygen rapidly restored cerebral blood flow, long-term sensorimotor deficit was assessed, raising concerns regarding post-resuscitation hyperemia [26]. In rat pups exposed to hypoxia–reoxygenation, using diaphragm electromyography, Bookatz et al. confirmed that the use of high oxygen concentrations delayed the onset of the diaphragmatic response as a consequence of spontaneous respiration [27].

Oxygen in excess alters lung permeability, causing an inflammatory response with the destruction of the alveolar–capillary barrier, augmented pulmonary permeability, and endothelial and epithelial cell death [27]. Moreover, the use of pure oxygen upon reoxygenation, causes damage to other organs, such as the brain, heart, lungs, and liver, as compared with the use of ambient air [28,29]. In addition, other studies with a similar animal models showed cell death in the brain cortex associated with high levels of oxygen [30]. Interestingly, hypoxic preconditioning before reoxygenation decreased the biomarkers of oxidative damage to the DNA in the brain tissue [31]. Hyperoxia also has the potential to alter genome activity via changes in the DNA methylome. Understanding the epigenetic potential of hyperoxia would enable further improvement of the therapeutic strategies for chronic lung disease [28]. Experimental studies in hypoxia–reoxygenation have been exquisitely summarized by Saugstad OD et al. in a recent review article [32].

Translating into the clinical setting, findings in animal experiments would provide more evidence supporting the observation that the fetal-to-neonatal transition under hypoxic conditions appears to be protective and that targeting high saturations too rapidly in premature infants just after birth should be avoided. However, although highly informative, the results of the experiments performed on rodents cannot be directly translated into the human physiology or pathophysiology. There are substantial differences in the maturation of rodents in the last phase of gestation compared with that of humans. Hence, rodents are born with very immature lung and brain development as compared with full-term neonates. However, there is no a direct correlation between extremely premature humans surviving in the neonatal intensive care unit and rat pups that are able to survive in the experimental setting.

## 4. Pathologic Conditions during the Fetal-to-Neonatal Transition in Newborn Infants

### 4.1. Perinatal Asphyxia

Perinatal asphyxia is characterized by intermittent periods of hypoxia ischemia that may cause irreversible damage to brain [33]. The pathophysiologic effects of hypoxic ischemia are complex and evolve over time. Hence, a primary phase corresponds to tissue hypoxia followed by a secondary phase characterized by partial recovery upon reoxygenation/reperfusion. The primary energy failure leads to significantly decreased ATP and increased lactate production. The inactivation of ATP-dependent ion pumps causes an excessive influx of sodium ions into the cytoplasm, instigating cell swelling and an inhibition of the synaptic recapturing of neurotransmitters, leading to hyperexcitability. On the other hand, the concomitant increase in intracellular calcium has evident harmful effects, leading to cerebral edema, ischemia, microvascular damage, and necrosis. The secondary energy failure phase occurs 6–48 h after the initial injury [34]. The mechanisms of secondary energy failure produce OS, excitotoxicity, inflammation, and mitochondrial dysfunction. Increased levels of free radicals cause damage to neuronal cell membranes and lead to necrosis or apoptosis [35]. OS is particularly harmful to the neonatal brain due to low concentrations of antioxidants and a high consumption of oxygen when transitioning from fetal to neonatal life [36]. One of the most relevant sources of free radicals that enhance cellular damage upon reperfusion is related to the conversion of xanthine dehydrogenase (XD) to xanthine oxidase (XO). In addition, ATP is converted into hypoxanthine, a purine substrate for XO. Upon reoxygenation XO converts hypoxanthine to uric acid and molecular oxygen to a superoxide and H_2_O_2_. During a hypoxic-ischemic state, the iron that was bound to proteins is released, which makes the free iron (Fe^2+^) available to react with peroxides and form free radicals [37] (Figure 1).

Several studies have described the molecular and metabolomic changes associated with asphyxia and resuscitation using different oxygen concentrations. In experimental studies, Saugstad et al. showed that hypoxanthine accumulation in different animal models of hypoxia directly depended on the duration and intensity of the hypoxia [38,39]. Moreover, in a piglet model of severe hypoxia, Solberg et al. showed changes in specific metabolites in the plasma metabolome [40,41]. In the same experimental model, Sánchez-Illana et al. showed the potential of choline and a panel of related metabolites to enhance the predictive accuracy of the intensity and duration of hypoxia as compared with plasma lactate [41]. Finally, Kuligowski et al. validated a “hypoxia metabolic score” that included choline, 6,8-dihydroxypurine, and hypoxanthine and was highly reliable in predicting the duration and intensity of hypoxia in a piglet model [42]. A study of intermediaries of the Krebs’ cycle in animal models showed that α-ketoglutarate, succinate, and fumarate levels decreased slower after reoxygenation with 100% O_2_ than with air. The results obtained during reoxygenation with high oxygen concentration revealed mitochondrial dysfunction [43]. Our study showed, for the first time ex vivo, that during reperfusion there is a transient shift from complex I-dependent oxidation of NADH towards complex II-linked oxidation of succinate in the brain mitochondria. As a consequence, electron transport in the electron transport complex is reversed, causing a substantial increase of ROS production [44].

In a series of randomized clinical studies, our group demonstrated the feasibility of resuscitating asphyxiated babies with ambient air instead of pure oxygen. Moreover, these studies also showed that using ambient air significantly reduced the markers of oxidative stress and damage [45,46,47]. In a meta-analysis that included over 2000 asphyxiated newborn infants, the use of air significantly reduced mortality [48]. The international guidelines for full-term newborn resuscitation were changed thereafter, and the initial inspired fraction of oxygen (FiO_2_) recommended for asphyxiated full-term babies is 0.21 [49].

### 4.2. Prematurity

Preterm infants have immature lungs and immune systems and are therefore predisposed to respiratory insufficiency that causes short- and long-term morbidities and neurodevelopmental impairment [50,51]. Based on previous experimental findings, Vento et al. [2] launched a clinical trial that randomized extremely premature infants to be initially stabilized with a lower (0.3) versus a higher (0.9) inspired fraction of oxygen (FiO_2_). Premature infants who received higher initial FiO_2_ exhibited higher OS as reflected by a significantly lower GSH/GSSG ratio, increased urinary elimination of isoprostanes, isofurans, and DNA and protein oxidation byproducts. Remarkably, babies with increased markers of OS had a higher incidence of BPD [2]. Ezaki et al. [52] found that premature infants who received pure oxygen in the delivery room had significantly higher total peroxides and lower redox potential/total hydroperoxides ratios than neonates who received lower oxygen loads. Tataranno et al. [53] found a significant increase in the plasma levels of advanced oxidative protein products and isoprostanes 12 h after birth in premature babies receiving 100% oxygen. Kapadia et al. compared, from a clinical and analytical perspective, the consequences of using 100% oxygen or lower FiO_2_’s in the delivery room in extremely premature infants [54]. Total hydroperoxides (TH), biological antioxidant potential (BAP), and the TH/BAP quotient were determined in cord blood and 1 h after birth. At 1 h after birth, TH/BAP was significantly higher in the premature infants ventilated initially with air. In addition, these babies needed fewer days of mechanical ventilation and had a lower incidence of BPD [54]. More recently, two randomized, controlled, blinded trials in babies having experienced <32 weeks of gestation compared the use of an initial FiO_2_ of 0.3 versus an initial FiO_2_ of 0.6 in the delivery room. The analytical results showed no significant differences in OS biomarkers or in incidence of BPD [55,56]. It should be underscored that after titrating FiO_2_ according to the patients’ clinical needs, both groups received a similar oxygen load during stabilization [55,56]. Our studies provided the evidence for a cautious oxygen supplementation in premature infants during postnatal stabilization that was reflected in the most recent international resuscitation guidelines [49].

## 5. Biomarkers of Oxidative Stress Employed in the Clinical Setting during the Neonatal Period

**Figure 2:**
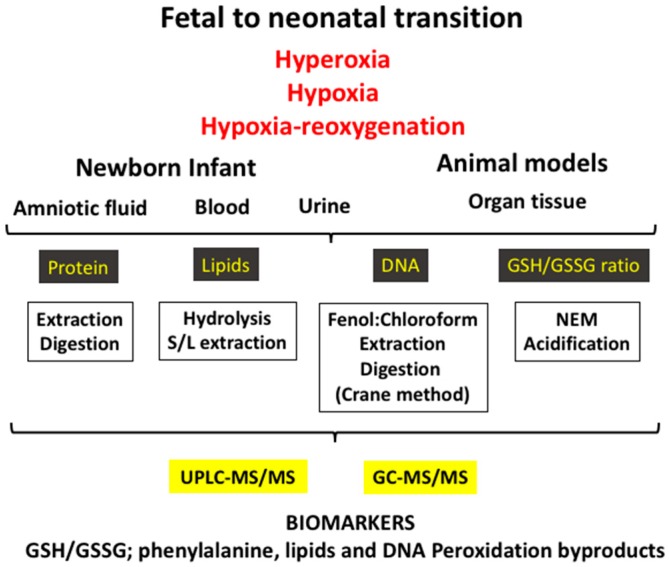
The fetal-to-neonatal transition increases exponentially the availability of oxygen to tissues, causing a physiological oxidative stress. However, under pathologic conditions (e.g., hyperoxia, hypoxia–reoxygenation, and hypoxia) a severe oxidative stress ensues that leads to pathologic conditions. Using biofluids (e.g., blood/plasma, urine, amniotic fluid) or tissue in experimental models and applying diverse laboratory techniques, an ample array of validated biomarkers of oxidative stress can be determined.

The biomarkers employed to evaluate OS are byproducts of oxidative damage to proteins, lipids, and nucleotides, and they can be measured in different fluids such as blood, urine, spinal fluid, or amniotic fluid or in different tissues in the experimental setting (Figure 2). Furthermore, low-molecular-weight thiols, namely the GSH/GSSG ratio, are other biomarkers, which are clear indicators of the redox status of the cell, and these are generally determined in the blood (Table 1).

### 5.1. Ratio of Reduced Glutathione to Oxidized Glutathione (GSH/GSSG)

The GSH/GSSG ratio is a reliable marker extensively used in clinical studies, because it gives a comprehensive picture of the cell redox status [2,45,46,47,55,56]. The major plasma aminothiols are GSH, cysteine, and homocysteine. Aminothiols interact through disulfide exchange and reduction–oxidation reactions regulating the cellular redox code as a consequence of most of the cell functions. The ratios of redox pairs—GSH/GSSG, Cysteine/Cystine, and Homocysteine/Homocystine—were proved to be useful for assessing OS [57].

Aminothiols in biological samples are measured using UPLC-MS/MS. The sample treatment is relatively simple. The samples are acidified with perchloric acid to remove the protein and separated by centrifugation, and the supernatant is analyzed. A specific characteristic in the sample treatment is the addition of N-ethylmaleimide (NEM) to avoid thiols oxidation. Our group has validated a method, which requires appropriate sample preparation and dilution, to obtain accurate and reproducible determinations [57].

In addition, the activity of antioxidant enzymes, such as SOD, CAT, and glutathione peroxidase (GPX), is used as an indirect marker of the response to a pro-oxidant aggression; however, it may fail to reflect oxidative damage to cellular structures (for a description and references see [23]).

Recently, our lab has developed a new method based on surface enhanced Raman spectroscopy (SERS) to easily analyze GSH. We have performed analytical monitoring with high reliability by using a silver colloid that enhances the GSH signal and allows for the accurate measurement of microvolumes (20 µL) of blood. This novel analytical tool has been validated and is extremely suitable, using a portable optical sensor device to perform point-of-care (POC) testing of OS levels in newborns [58].

### 5.2. Protein Oxidation

Proteins represent an extensive target for ROS and RNS generated under normal or OS conditions. Several amino acidic residues can undergo oxidative modifications, including the oxidation of sulfur-containing amino acid residues, the hydroxylation of aromatic groups, the nitration of tyrosine residues, the chlorination of aromatic groups, or the conversion of some amino acid residues to carbonyl derivatives. In the neonatal period, one of the main biomarkers measured is the oxidation of phenylalanine (Phe). The oxidation of Phe is produced by hydroxyl (OH) radical attack, which converts the Phe to ortho-tyrosine (o-Tyr) or meta-tyrosine (m-Tyr). In addition, other tyrosine-derived byproducts, such as 3-nitrotyrosine (3NO_2_-Tyr) and 3-chlorotyrosine (3Cl-Tyr), are also useful as biomarkers of nitrosative stress and inflammation, respectively. In the presence of peroxynitrite (ONOO^−^), p-Tyr can be converted into 3NO_2_-Tyr, a specific biomarker for protein nitration [59]. Protein nitration and RNS signaling play a role in cell functions such as inflammatory response and apoptosis [60]. Similarly, 3Cl-Tyr, formed by the attack of hypochlorous acid on p-Tyr through the action of the enzyme myeloperoxidase (MPO), is considered to be a useful inflammatory biomarker [61].

The results of these determinations are expressed as ratios, such as the o-Tyr/Phe, m-Tyr/Phe, 3NO_2_-Tyr/p-Tyr, and 3Cl-Tyr/p-Tyr ratios. These markers have been validated and extensively applied in the clinical setting in different human biofluid matrices, such as amniotic fluid, urine, plasma, cerebrospinal fluid, or human milk in the newborn period [2,62,63,64].

### 5.3. DNA Oxidation

Under physiological conditions, oxidative DNA damage is continuously being produced, but enzymatic DNA repair mechanisms are capable of reverting the situation, maintaining a low, steady state of oxidative damage [65]. Therefore, DNA oxidation may be secondarily aggravated by oxidative protein damage to the repair mechanisms.

The oxidation of DNA components by ROS is the major source of induced DNA damage, leading to several types of modifications, including nucleotide oxidation, strand breakage, loss of bases, and adduct formation. Hydroxyl radicals may attack the deoxyribose phosphate backbone, as well as the nitrogenous bases of DNA nucleotides, generating a broad variety of base and sugar modification products [66].

The main damage for OS to DNA is generally produced by guanosine base oxidation products, such as 7,8-hydroxy-2′deoxyguanosine (8-oxodG or 8-OHdG), which can be quantitatively measured in urine, plasma, CSF, etc., by UPLC-MS/MS [31]. The results are expressed as the 8-oxodG/2dG ratio [65,66,67,68].

### 5.4. Lipid Peroxidation

Lipids are readily attacked by free radicals, and lipid peroxidation byproducts reflect oxidative damage in a variety of biological samples. Lipids peroxidation may occur through enzymatic reactions, catalyzed by lipoxygenase (LOX) and cyclooxygenase (COX), which oxidize arachidonic acid (AA) into prostaglandins, prostacyclin, thromboxane, leukotrienes, and lipoxins [69]. All the omega-6 and omega-3 polyunsaturated fatty acids (PUFA) can undergo such enzymatic oxidation, leading to protectins, maresins, and dihydroxy-PUFA (diHPUFA) [70]. The neonatal markers of the peroxidation of PUFA are isoprostanes (IsoPs), isofurans (IsoFs), dihomo-isoprostanes (Dihomo-IsoPs), neuroprostanes (NeuroPs), and neurofurans (NeuroFs). Both are formed through the non-enzymatic free-radical oxidation of AA and other PUFAs and are chemically stable compounds. These analytes have been detected in fluids and tissues such as brain tissue, which has a high content of unsaturated lipids susceptible to OS [71,72,73]. Kuligowski et al. found that premature infants with high urinary IsoFs levels in the first days after birth are at higher risk of developing chronic lung disease [74]. Cháfer-Pericas et al. validated a method for identifying plasma lipid peroxidation biomarkers [75] and established a correlation between 8-iso-15(R)-PGF2 in cord blood with a degree asphyxia to improve diagnostic indices [76]. Barden et al. reported a significant increase of NeuroPs in cord blood, which was inversely correlated with maternal IsoPs, IsoFs, and NeuroPs and could be used estimate birth weight at delivery [77]. On the other hand, we have also validated analytical methods to determine docosahexaenoic acid (DHA) and adrenic acid (AdA). Both are important structural components of the central nervous system. During lipid peroxidation, NeuroPs and NeuroFs arise from DHA oxidation, and Dihomo-IsoPs are generated from AdA. NeuroPs and NeuroFs are highly sensitive and specific markers of neuronal oxidative damage [78].

Several sensitive analytical techniques, such as GC-MS/MS or UPLC-MS/MS, allow for the determination of these compounds in the plasma and tissue matrix, but the interpretation of the results involve many difficulties because of the large number of metabolites, including highly similar structural isomers, sharing physicochemical properties, and the chromatographic behavior.

Our group has contributed to the expansion of these methods in the newborn period. Kuligowski et al. [69,74] developed a UPLC-MS/MS method for recording the profiles of the relative contents of IsoPs, IsoFs, NeuroPs, and NeuroFs in a total of 536 urine samples during the first 4 weeks of life in 184 premature infants who did not develop any free radical-associated conditions during the neonatal period. In addition, Sánchez-Illana et al. developed and validated a comprehensive UPLC-MS/MS approach for the quantitative profiling of 28 isoprostanoids in 150 plasmas from newborns with hypoxic-ischemic encephalopathy, covering a broad range of lipid peroxidation product classes [78]. The access to a large sample set of this especially vulnerable population has allowed us to establish a time frame during which lipid peroxidation byproducts could be quantified and compared to normality ranges [69,78]. The applicability of these new methods to non-invasively obtained samples, such as urine [69] or saliva [79], opens a new window for the precise evaluation of oxidative stress-related conditions in the perinatal period.

## 6. Conclusions

Oxidative stress-related conditions are extremely frequent in the early neonatal period and contribute to increase mortality and morbidities that may last lifelong. The search for new biomarkers, which serve as diagnostic tools, by using accurate analytical methods on matrices that can be obtained without causing pain or damage to vulnerable newborn infants has become more and more necessary to guide interventions and monitor results. Cot-side oxidative stress evaluation using microsamples and providing rapid results will permit earlier diagnosis, improve our understanding of the consequences of our interventions, avoid possible complications, and evaluate the degree of success of the applied therapies.

## Figures and Tables

**Table 1:** Summary of the most relevant biomarkers used in neonatal clinical research.

Oxidative Biomarker	Target Biomolecule	Modification	Biological Sampling
GSH/GSSG Cysteine/Cystine Homocysteine/Homocystine	Antioxidant	Redox status	Umbilical cord blood/whole blood/tissue
o-Tyr/Phe ratio m-Tyr/Phe ratio 3NO_2_-Tyr/p-Tyr ratio 3Cl-Tyr/p-Tyr ratio	Protein	Tyrosine Hydroxylation Tyrosine nitration Tyrosine chlorination	Urine/plasma/milk/tissue
8oxodG (8oxodG/2dG ratio)	DNA	Hydroxylation DNA nucleotides	Urine/plasma/amniotic fluid/tissue
IsoP	Lipids	AA peroxidation	Urine/plasma/amniotic fluid/tissue
Dihomo-IsoP	Lipids	AdA peroxidation	Urine/plasma/tissue
IsoF	Lipids	AA peroxidation	Urine/plasma/tissue
NeuroP	Lipids	DHA peroxidation	Urine/plasma/tissue
NeuroF	Lipids	DHA peroxidation	Urine/plasma/tissue

o-Tyr/Phe = orto-tyrosine/Phenylalanine ratio; m-Tyr/Phe = meta-tyrosine/Phenylalanine ratio; 3NO_2_-Tyr/p-Tyr = nitrotyrosine/para-tyrosine ratio; 3-Cl-tyr/p-Tyr ratio = 3 chlortyrosine/para-Tyrosine ratio. Isop = Isoprostanes; Dihomo-Isop = Dihomo-Isoprostanes; IsoF = isofurans; NeuroP = Neuroprostanes; NeuroF = neurofurans; Ada = adrenic acid; AA = arachidonic acid; DHA = docosahexanoic acid.

## References

[1] Morton S.U., Brodsky D. (2016). Fetal Physiology and the Transition to Extrauterine Life. Clin. Perinatol..

[2] Vento M., Moro M., Escrig R., Arruza L., Villar G., Izquierdo I., Roberts L.J., Arduini A., Escobar J.J., Sastre J. (2009). Preterm resuscitation with low oxygen causes less oxidative stress, inflammation, and chronic lung disease. Pediatrics.

[3] Negro S., Benders M.J.N.L., Tataranno M.L., Coviello C., de Vries L.S., van Bel F., Groenendaal F., Longini M., Proietti F., Belvisi E. (2017). Oxygen and oxidative stress in the perinatal period. Redox Biol..

[4] Campbell I. (2017). Intermediary metabolism. Anaesth. Intensiv. Care Med..

[5] Lobo V., Patil A., Phatak A., Chandra N. (2010). Free radicals, antioxidants and functional foods: Impact on human health. Pharmacogn. Rev..

[6] Circu M.L., Aw T.Y. (2010). Reactive oxygen species, cellular redox systems and apoptosis. Free Radic. Biol. Med..

[7] Beharry K.D., Cai C.L., Valencia G.B., Valencia A.M., Lazzaro D.R., Bany-Mohammed F., Aranda J.V. (2017). Neonatal Intermittent Hypoxia, Reactive Oxygen Species, and Oxygen-Induced Retinopathy. React. Oxyg. Species.

[8] Kalogeris T., Bao Y., Korthuis R.J. (2014). Mitochondrial reactive oxygen species: A double edged sword in ischemia/reperfusion vs. preconditioning. Redox Biol..

[9] Sies H., Berndt C., Jones D.P. (2017). Oxidative Stress. Annu. Rev. Biochem..

[10] Trachootham D., Zhou Y., Zhang H., Demizu Y., Chen Z., Pelicano H., Chiao P.J., Achanta G., Arlinghaus R.B., Liu J. (2006). Selective killing of oncogenically transformed cells through a ROS-mediated mechanism by beta-phenylethyl isothiocyanate. Cancer Cell.

[11] Schieber M., Chandel N.S. (2014). ROS Function in Redox Signaling and Oxidative Stress. Curr. Biol..

[12] Bubb K.J., Birgisdottir A.B., Tang O., Hansen T., Figtree G.A. (2017). Redox modification of caveolar proteins in the cardiovascular system-role in cellular signaling and disease. Free Radic. Biol. Med..

[13] Jones D.P. (2010). Redox sensing: Orthogonal control in cell cycle and apoptosis signalling. J. Intern. Med..

[14] Jones D.P., Sies H. (2015). The Redox Code. Antioxid. Redox Signal..

[15] Baik N., Urlesberger B., Schwaberger B., Schmölzer G.M., Mileder L., Avian A., Pichler G. (2015). Reference Ranges for Cerebral Tissue Oxygen Saturation Index in Term Neonates during Immediate Neonatal Transition after Birth. Neonatology.

[16] Yigit M.B., Kowalski W.J., Hutchon D.J.R., Pekkan K. (2015). Transition from fetal to neonatal circulation: Modeling the effect of umbilical cord clamping. J. Biomech..

[17] Dawson J.A., Kamlin C.O.F., Vento M., Wong C., Cole T.J., Donath S.M., Davis P.G., Morley C.J. (2010). Defining the reference range for oxygen saturation for infants after birth. Pediatrics.

[18] Agarwal A., Aponte-Mellado A., Premkumar B.J., Shaman A., Gupta S. (2012). The effects of oxidative stress on female reproduction: A review. Reprod. Biol. Endocrinol. RBE.

[19] Shim S.-Y., Kim H.-S. (2013). Oxidative stress and the antioxidant enzyme system in the developing brain. Korean J. Pediatr..

[20] Vento M., Aguar M., Escobar J., Arduini A., Escrig R., Brugada M., Izquierdo I., Asensi M.A., Sastre J., Saenz P. (2009). Antenatal steroids and antioxidant enzyme activity in preterm infants: Influence of gender and timing. Antioxid. Redox Signal..

[21] Viña J., Vento M., García-Sala F., Puertes I.R., Gascó E., Sastre J., Asensi M., Pallardó F.V. (1995). L-cysteine and glutathione metabolism are impaired in premature infants due to cystathionase deficiency. Am. J. Clin. Nutr..

[22] Pallardo F.V., Sastre J., Asensi M., Rodrigo F., Estrela J.M., Viña J. (1991). Physiological changes in glutathione metabolism in foetal and newborn rat liver. Biochem. J..

[23] Maltepe E., Saugstad O.D. (2009). Oxygen in health and disease: Regulation of oxygen homeostasis—Clinical implications. Pediatr. Res..

[24] Sastre J., Asensi M., Rodrigo F., Pallardó F.V., Vento M., Viña J. (1994). Antioxidant administration to the mother prevents oxidative stress associated with birth in the neonatal rat. Life Sci..

[25] Gelfand S.L., Vento M., Sastre J., Lust W.D., Smith M.A., Perry G., Walsh M., Martin R. (2008). A new model of oxidative stress in rat pups. Neonatology.

[26] Presti A.L., Kishkurno S.V., Slinko S.K., Randis T.M., Ratner V.I., Polin R.A., Ten V.S. (2006). Reoxygenation with 100% oxygen versus room air: Late neuroanatomical and neurofunctional outcome in neonatal mice with hypoxic-ischemic brain injury. Pediatr. Res..

[27] Bookatz G.B., Mayer C.A., Wilson C.G., Vento M., Gelfand S.L., Haxhiu M.A., Martin R.J. (2007). Effect of supplemental oxygen on reinitiation of breathing after neonatal resuscitation in rat pups. Pediatr. Res..

[28] Saugstad O.D. (2010). Oxygen and oxidative stress in bronchopulmonary dysplasia. J. Perinat. Med..

[29] Bhandari V. (2010). Hyperoxia-derived lung damage in preterm infants. Semin. Fetal Neonatal Med..

[30] Yee M., Cohen E.D., Domm W., Porter G.A., McDavid A.N., O’Reilly M.A. (2018). Neonatal hyperoxia depletes pulmonary vein cardiomyocytes in adult mice via mitochondrial oxidation. Am. J. Physiol. Lung Cell. Mol. Physiol..

[31] Torres-Cuevas I., Aupi M., Asensi M.A., Vento M., Ortega Á., Escobar J. (2017). 7,8-hydroxy-2′-deoxyguanosine/2′-deoxiguanosine ratio determined in hydrolysates of brain DNA by ultrachromatrography coupled to tandem mass spectrometry. Talanta.

[32] Saugstad O.D., Sejersted Y., Solberg R., Wollen E.J., Bjørås M. (2012). Oxygenation of the Newborn: A Molecular Approach. Neonatology.

[33] Merchant N., Azzopardi D. (2015). Early predictors of outcome in infants treated with hypothermia for hypoxic-ischaemic encephalopathy. Dev. Med. Child Neurol..

[34] Allen K.A., Brandon D.H. (2011). Hypoxic Ischemic Encephalopathy: Pathophysiology and Experimental Treatments. Newborn Infant Nurs. Rev. NAINR.

[35] Odeh M. (1991). The role of reperfusion-induced injury in the pathogenesis of the crush syndrome. N. Engl. J. Med..

[36] Buonocore G., Groenendaal F. (2007). Anti-oxidant strategies. Semin. Fetal Neonatal Med..

[37] Torres-Cuevas I., Cernada M., Nuñez A., Escobar J., Kuligowski J., Chafer-Pericas C., Vento M. (2016). Oxygen Supplementation to Stabilize Preterm Infants in the Fetal to Neonatal Transition: No Satisfactory Answer. Front. Pediatr..

[38] Saugstad O.D. (1975). Hypoxanthine as a measurement of hypoxia. Pediatr. Res..

[39] Saugstad O.D., Aasen A.O. (1980). Plasma hypoxanthine concentrations in pigs. A prognostic aid in hypoxia. Eur. Surg. Res. Eur. Chir. Forsch. Rech. Chir. Eur..

[40] Solberg R., Kuligowski J., Pankratov L., Escobar J., Quintás G., Lliso I., Sánchez-Illana Á., Saugstad O.D., Vento M. (2016). Changes of the plasma metabolome of newly born piglets subjected to postnatal hypoxia and resuscitation with air. Pediatr. Res..

[41] Sánchez-Illana Á., Solberg R., Lliso I., Pankratov L., Quintás G., Saugstad O.D., Vento M., Kuligowski J. (2017). Assessment of phospholipid synthesis related biomarkers for perinatal asphyxia: A piglet study. Sci. Rep..

[42] Kuligowski J., Solberg R., Sánchez-Illana Á., Pankratov L., Parra-Llorca A., Quintás G., Saugstad O.D., Vento M. (2017). Plasma metabolite score correlates with Hypoxia time in a newly born piglet model for asphyxia. Redox Biol..

[43] Sahni P.V., Zhang J., Sosunov S., Galkin A., Niatsetskaya Z., Starkov A., Brookes P.S., Ten V.S. (2018). Krebs cycle metabolites and preferential succinate oxidation following neonatal hypoxic-ischemic brain injury in mice. Pediatr. Res..

[44] Sánchez-Illana Á., Núñez-Ramiro A., Cernada M., Parra-Llorca A., Valverde E., Blanco D., Moral-Pumarega M.T., Cabañas F., Boix H., Pavón A. (2017). Evolution of Energy Related Metabolites in Plasma from Newborns with Hypoxic-Ischemic Encephalopathy during Hypothermia Treatment. Sci. Rep..

[45] Vento M., Asensi M., Sastre J., García-Sala F., Pallardó F.V., Viña J. (2001). Resuscitation with Room Air Instead of 100% Oxygen Prevents Oxidative Stress in Moderately Asphyxiated Term Neonates. Pediatrics.

[46] Vento M., Asensi M., Sastre J., Lloret A., García-Sala F., Viña J. (2003). Oxidative stress in asphyxiated term infants resuscitated with 100% oxygen. J. Pediatr..

[47] Vento M., Sastre J., Asensi M.A., Viña J. (2005). Room-air resuscitation causes less damage to heart and kidney than 100% oxygen. Am. J. Respir. Crit. Care Med..

[48] Saugstad O.D., Ramji S., Soll R.F., Vento M. (2008). Resuscitation of newborn infants with 21% or 100% oxygen: An updated systematic review and meta-analysis. Neonatology.

[49] Perlman J.M., Wyllie J., Kattwinkel J., Wyckoff M.H., Aziz K., Guinsburg R., Kim H.-S., Liley H.G., Mildenhall L., Simon W.M. (2015). Part 7: Neonatal Resuscitation: 2015 International Consensus on Cardiopulmonary Resuscitation and Emergency Cardiovascular Care Science with Treatment Recommendations. Pediatrics.

[50] Vento M. (2014). Oxygen supplementation in the neonatal period: Changing the paradigm. Neonatology.

[51] Saigal S., Doyle L.W. (2008). An overview of mortality and sequelae of preterm birth from infancy to adulthood. Lancet.

[52] Ezaki S., Suzuki K., Kurishima C., Miura M., Weilin W., Hoshi R., Tanitsu S., Tomita Y., Takayama C., Wada M. (2009). Resuscitation of preterm infants with reduced oxygen results in less oxidative stress than resuscitation with 100% oxygen. J. Clin. Biochem. Nutr..

[53] Tataranno M.L., Oei J.L., Perrone S., Wright I.M., Smyth J.P., Lui K., Tarnow-Mordi W.O., Longini M., Proietti F., Negro S. (2015). Resuscitating preterm infants with 100% oxygen is associated with higher oxidative stress than room air. Acta Paediatr..

[54] Kapadia V.S., Chalak L.F., Sparks J.E., Allen J.R., Savani R.C., Wyckoff M.H. (2013). Resuscitation of preterm neonates with limited versus high oxygen strategy. Pediatrics.

[55] Rook D., Schierbeek H., Vento M., Vlaardingerbroek H., van der Eijk A.C., Longini M., Buonocore G., Escobar J., van Goudoever J.B., Vermeulen M.J. (2014). Resuscitation of preterm infants with different inspired oxygen fractions. J. Pediatr..

[56] Aguar M., Izquierdo M., Brugada M. Preterm babies randomly assigned to be blindly resuscitated with higher (60%) vs. lower (30%) initial FIO2: Effects on oxidative stress and mortality. Proceedings of the EAPS.

[57] Escobar J., Sánchez-Illana Á., Kuligowski J., Torres-Cuevas I., Solberg R., Garberg H.T., Huun M.U., Saugstad O.D., Vento M., Cháfer-Pericás C. (2016). Development of a reliable method based on ultra-performance liquid chromatography coupled to tandem mass spectrometry to measure thiol-associated oxidative stress in whole blood samples. J. Pharm. Biomed. Anal..

[58] Sánchez-Illana Á., Mayr F., Cuesta-García D., Piñeiro-Ramos J.D., Cantarero A., Guardia M.D.L., Vento M., Lendl B., Quintás G., Kuligowski J. (2018). On-Capillary Surface-Enhanced Raman Spectroscopy: Determination of Glutathione in Whole Blood Microsamples. Anal. Chem..

[59] Tsikas D. (2012). Analytical methods for 3-nitrotyrosine quantification in biological samples: The unique role of tandem mass spectrometry. Amino Acids.

[60] Franco M.C., Estévez A.G. (2014). Tyrosine nitration as mediator of cell death. Cell. Mol. Life Sci. CMLS.

[61] Torres-Cuevas I., Kuligowski J., Cárcel M., Cháfer-Pericás C., Asensi M., Solberg R., Cubells E., Nuñez A., Saugstad O.D., Vento M. (2016). Protein-bound tyrosine oxidation, nitration and chlorination by-products assessed by ultraperformance liquid chromatography coupled to tandem mass spectrometry. Anal. Chim. Acta.

[62] Solberg R., Andresen J.H., Escrig R., Vento M., Saugstad O.D. (2007). Resuscitation of hypoxic newborn piglets with oxygen induces a dose-dependent increase in markers of oxidation. Pediatr. Res..

[63] Ledo A., Arduini A., Asensi M.A., Sastre J., Escrig R., Brugada M., Aguar M., Saenz P., Vento M. (2009). Human milk enhances antioxidant defenses against hydroxyl radical aggression in preterm infants. Am. J. Clin. Nutr..

[64] Escobar J., Teramo K., Stefanovic V., Andersson S., Asensi M.A., Arduini A., Cubells E., Sastre J., Vento M. (2013). Amniotic fluid oxidative and nitrosative stress biomarkers correlate with fetal chronic hypoxia in diabetic pregnancies. Neonatology.

[65] Valavanidis A., Vlachogianni T., Fiotakis C. (2009). 8-hydroxy-2′-deoxyguanosine (8-OHdG): A critical biomarker of oxidative stress and carcinogenesis. J. Environ. Sci. Health Part C Environ. Carcinog. Ecotoxicol. Rev..

[66] Ambroz A., Vlkova V., Rossner P., Rossnerova A., Svecova V., Milcova A., Pulkrabova J., Hajslova J., Veleminsky M., Solansky I. (2016). Impact of air pollution on oxidative DNA damage and lipid peroxidation in mothers and their newborns. Int. J. Hyg. Environ. Health.

[67] Loft S., Danielsen P., Løhr M., Jantzen K., Hemmingsen J.G., Roursgaard M., Karotki D.G., Møller P. (2012). Urinary excretion of 8-oxo-7,8-dihydroguanine as biomarker of oxidative damage to DNA. Arch. Biochem. Biophys..

[68] Barregard L., Møller P., Henriksen T., Mistry V., Koppen G., Rossner P., Sram R.J., Weimann A., Poulsen H.E., Nataf R. (2013). Human and methodological sources of variability in the measurement of urinary 8-oxo-7,8-dihydro-2′-deoxyguanosine. Antioxid. Redox Signal..

[69] Kuligowski J., Escobar J., Quintás G., Lliso I., Torres-Cuevas I., Nuñez A., Cubells E., Rook D., van Goudoever J.B., Vento M. (2014). Analysis of lipid peroxidation biomarkers in extremely low gestational age neonate urines by UPLC-MS/MS. Anal. Bioanal. Chem..

[70] Serhan C.N., Chiang N., Dalli J. (2018). New pro-resolving n-3 mediators bridge resolution of infectious inflammation to tissue regeneration. Mol. Aspects Med..

[71] Milne G.L., Dai Q., Roberts L.J. (2015). The isoprostanes—25 years later. Biochim. Biophys. Acta.

[72] Solberg R., Longini M., Proietti F., Vezzosi P., Saugstad O.D., Buonocore G. (2012). Resuscitation with supplementary oxygen induces oxidative injury in the cerebral cortex. Free Radic. Biol. Med..

[73] Galano J.-M., Lee Y.Y., Oger C., Vigor C., Vercauteren J., Durand T., Giera M., Lee J.C. (2017). Isoprostanes, neuroprostanes and phytoprostanes: An overview of 25 years of research in chemistry and biology. Prog. Lipid Res..

[74] Kuligowski J., Aguar M., Rook D., Lliso I., Torres-Cuevas I., Escobar J., Quintás G., Brugada M., Sánchez-Illana Á., van Goudoever J.B. (2015). Urinary Lipid Peroxidation Byproducts: Are They Relevant for Predicting Neonatal Morbidity in Preterm Infants?. Antioxid. Redox Signal..

[75] Cháfer-Pericás C., Torres-Cuevas I., Sanchez-Illana A., Escobar J., Kuligowski J., Solberg R., Garberg H.T., Huun M.U., Saugstad O.D., Vento M. (2016). Development of a reliable analytical method to determine lipid peroxidation biomarkers in newborn plasma samples. Talanta.

[76] Cháfer-Pericas C., Cernada M., Rahkonen L., Stefanovic V., Andersson S., Vento M. (2016). Preliminary case control study to establish the correlation between novel peroxidation biomarkers in cord serum and the severity of hypoxic ischemic encephalopathy. Free Radic. Biol. Med..

[77] Barden A.E., Corcoran T.B., Mas E., Durand T., Galano J.-M., Roberts L.J., Paech M., Muchatuta N.A., Phillips M., Mori T.A. (2012). Is There a Role for Isofurans and Neuroprostanes in Pre-Eclampsia and Normal Pregnancy?. Antioxid. Redox Signal..

[78] Sánchez-Illana Á., Thayyil S., Montaldo P., Jenkins D., Quintás G., Oger C., Galano J.-M., Vigor C., Durand T., Vento M. (2017). Novel free-radical mediated lipid peroxidation biomarkers in newborn plasma. Anal. Chim. Acta.

[79] Peña-Bautista C., Carrascosa-Marco P., Oger C., Vigor C., Galano J., Durand T., Baquero M., López-Nogueroles M., Vento M., García-Blanco A.C. (2018). Validated analytical method to determine new salivary lipid peroxidation compounds as potential neurodegenerative biomarkers. J. Pharm. Biomed. Anal..

